# Magnetic resonance imaging of pulmonary hypertension

**DOI:** 10.1007/s00247-024-06099-w

**Published:** 2024-11-15

**Authors:** Christian Johannes Kellenberger

**Affiliations:** University Children’s Hospital Zurich – Eleonore Foundation, Lenggstrasse 30, CH-8008 Zürich, Switzerland

**Keywords:** Children, Congenital heart disease, Magnetic resonance imaging, Pulmonary hypertension, Pulmonary vascular disease, Time-resolved dynamic contrast-enhanced angiography

## Abstract

Pulmonary hypertension is a rare but serious disease in children with potentially poor prognosis in the long term. Direct measurement of increased pressure in the pulmonary circulation requires right-heart catheterisation. Echocardiography is the imaging modality commonly used for suggesting the presence of pulmonary hypertension and estimating its severity. Recognition of structural and haemodynamic signs of increased pulmonary arterial pressure and pressure overload of the right ventricle at magnetic resonance (MR) imaging may contribute to the diagnosis of pulmonary hypertension and management of these patients. In this article, the structural, functional, and haemodynamic cardiovascular MR findings of paediatric pulmonary hypertension are reviewed. Typical diagnostic MR scenarios in children with suspected pulmonary hypertension or in children with disease associated with pulmonary hypertension are presented and discussed.

**Graphical Abstract:**

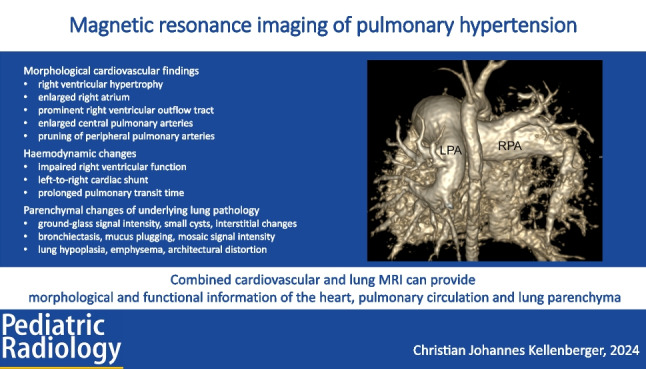

## Introduction

Pulmonary hypertension is characterised by elevated pulmonary arterial pressure, and increased pulmonary vascular resistance and pressure overload to the right heart. The most recent definition of precapillary pulmonary arterial hypertension is an increased mean pulmonary arterial pressure > 20 mmHg and increased pulmonary vascular resistance ≥ 3 Wood units as measured by right-heart catheterisation [1].

Right-heart catheterisation is the only method that allows direct measurement of the pulmonary artery pressure. Doppler echocardiography and magnetic resonance (MR) can be used to estimate the pressure in the pulmonary arteries and right heart. Further, MR can provide characteristic imaging findings for pulmonary hypertension and the underlying cardiovascular and pulmonary diseases.

Transthoracic echocardiography is used in patients with suspected pulmonary hypertension for estimating the systolic pulmonary arterial pressure and detecting additional signs that may indicate the presence of pulmonary hypertension [2]. The pressure in the main pulmonary artery and right ventricle is estimated by the modified Bernoulli equation from the regurgitation velocities over the pulmonary and tricuspid valves. A recent umbrella review of systematic reviews and meta-analyses published between 2010 and 2020 summarised that echocardiography has a high sensitivity but relatively low specificity in detecting moderate to severe pulmonary hypertension [3].

Although MR is not widely recommended for initial imaging of patients with suspected pulmonary hypertension [2], standard MR sequences reveal the same characteristic imaging findings as chest radiography, echocardiography, and chest computed tomography (CT) for diagnosing moderate to severe pulmonary hypertension. In addition, cardiac MR is the preferred modality for assessing functional abnormalities of the right ventricle. Flow-sensitive MR sequences and MR lung perfusion imaging can provide haemodynamic information of the pulmonary circulation that may allow for more sensitive detection of mild and asymptomatic pulmonary hypertension which potentially goes undetected by echocardiography.

In this article, structural and haemodynamic MR findings (Table 1) of pulmonary hypertension are reviewed. Typical clinical scenarios illustrating how MR may contribute to the diagnosis of pulmonary hypertension in children are presented.

**Table 1:** Cardiovascular and lung magnetic resonance findings in paediatric pulmonary hypertension

Pulmonary vasculature	Enlarged pulmonary trunk (increased MPA to AAO diameter ratio) Enlarged branch pulmonary arteries Pruning of peripheral pulmonary arteries Pulmonary vein stenosis or occlusion
Heart	Right ventricular hypertrophy and dilatation Leftward bowing of interventricular septum Tricuspid valve insufficiency Decreased right ventricular ejection fraction
Haemodynamics	Prolonged pulmonary transit times (increased pulmonary vascular resistance) Increased pulmonary to systemic blood flow ratio (left-to-right shunt) Decreased pulmonary to systemic blood flow ratio (Eisenmenger syndrome) Decreased blood flow to one lung (ipsilateral pulmonary vein stenosis or occlusion) Flow vortices in dilated pulmonary trunk Decreased pulmonary artery flow velocity and volume Decreased distension, pulsatility, wall shear stress, and kinetic energy in pulmonary trunk and branch pulmonary arteries
Lungs	Ground-glass intensity (ChILD, pulmonary veno-occlusive disease) Pulmonary oedema with thickened interlobular septae (idiopathic pulmonary hypertension, pulmonary veno-occlusive disease) Emphysema, thin-walled air-filled cysts (ChILD) Architectural distortion, inhomogeneous aeration and perfusion (BPD) Mosaic intensity and perfusion defects (perfusion abnormality or small airways disease – i.e. bronchiolitis obliterans, CF, PCD) Bronchiectasis, bronchial wall thickening, mucus plugging (CF, PCD) Hypoplasia

*AAO* ascending aorta, *BPD* bronchopulmonary dysplasia, *ChILD* childhood interstitial lung disease, *CF* cystic fibrosis, *MPA* main pulmonary artery, *PCD* primary ciliary dyskinesia

## Structural and functional cardiovascular magnetic resonance findings of pulmonary hypertension

**Figure 1:**
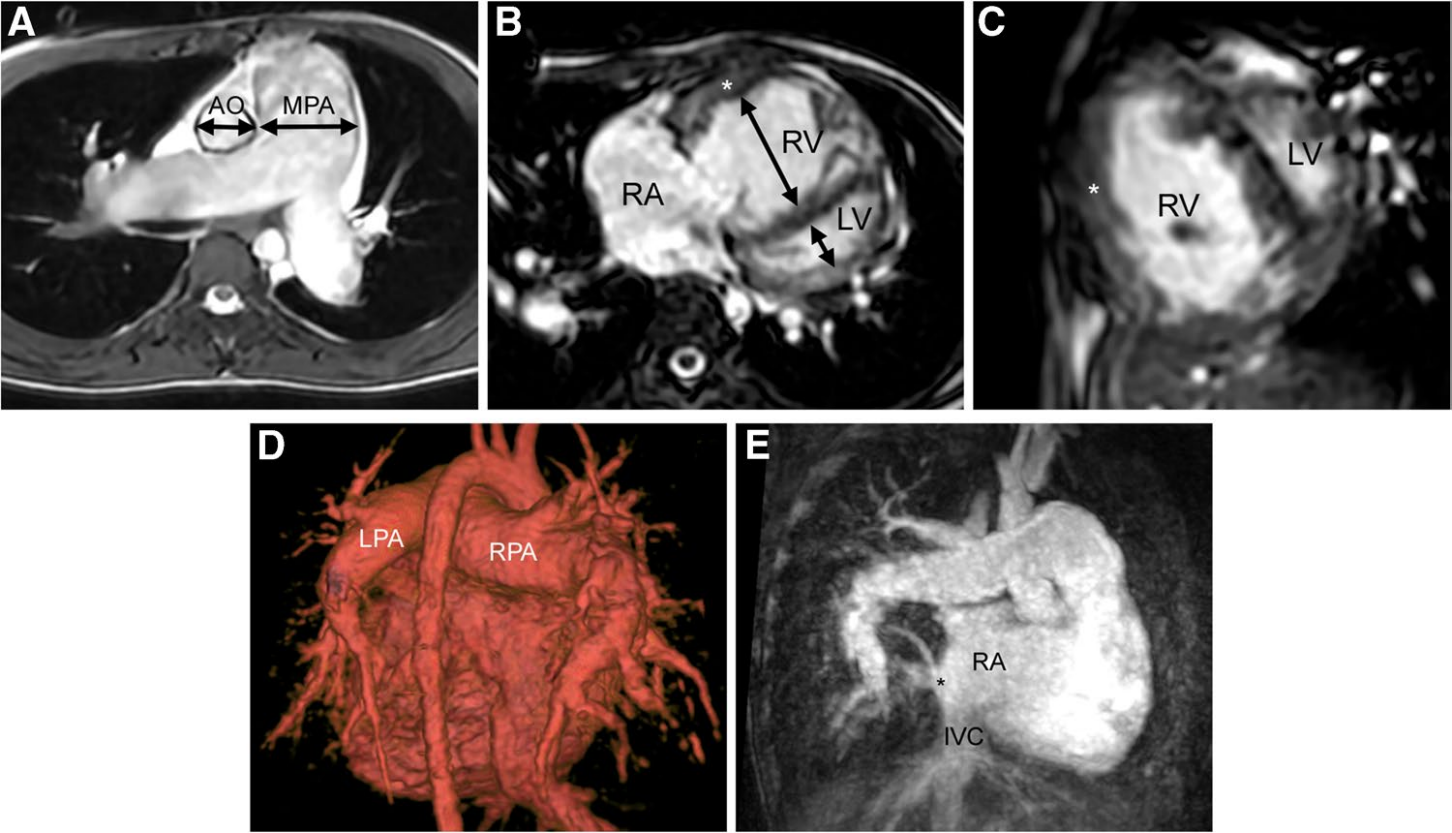
A 10-year-old girl with partial anomalous connection of the right lower pulmonary vein to the right atrium with imaging findings of pulmonary hypertension (images courtesy of Kantha Bopha Children’s Hospital, Phnom Penh, Cambodia). **a** Axial static steady-state free-precession (SSFP) localiser image shows a dilated pulmonary trunk and branch pulmonary arteries. The pulmonary artery (MPA) to ascending aorta (AO) diameter ratio is increased (34 mm/19 mm = 1.8). **b**, **c** Axial (**b**) and short-axis (**c**) end-systolic SSFP cine images show a dilated and hypertrophic right ventricle with increased diameters of the right ventricle (RV) exceeding that of the left ventricle (LV) (*arrows* in (**b**)—RV diameter > LV diameter), leftward bowing of the interventricular septum, and thickened right ventricular myocardium (*asterisk*). **d** Posterior volume-rendered, contrast-enhanced angiographic image illustrates dilatation of the central pulmonary arteries (RPA, LPA) with pruning of the peripheral pulmonary arteries. **e** Coronal-oblique contrast-enhanced angiographic image shows the anomalous connection of the left lower pulmonary vein (*asterisk*) to the right atrium (RA) above the junction to the inferior vena cava (IVC)

The cardiovascular findings for pulmonary hypertension are enlargement of the central pulmonary arteries, pruning of the peripheral pulmonary arteries, and dilated right cardiac chambers with hypertrophy of the right ventricular myocardium (Fig. 1).

In adults, a main pulmonary artery diameter of more than 30 mm and an increased ratio of the main pulmonary artery and ascending aorta diameters (MPA/AAO > 1) have been shown to be accurate and easily determinable signs for pulmonary hypertension in routine cross-sectional imaging [4].

In children, normal size-dependent diameters of the main pulmonary artery are available for both contrast-enhanced MR angiography [5] and CT [6]. Main pulmonary artery diameter thresholds that suggest pulmonary hypertension have been established from paediatric reference curves [6]. In children, the main pulmonary artery diameter can be slightly larger than the ascending aorta diameter [6, 7]. Therefore, an MPA/AAO diameter ratio > 1 should not be used as a paediatric sign for pulmonary hypertension. Independent of age, a paediatric MPA/AAO diameter ratio of 1.3 has been shown to have a positive likelihood ratio of 34 (positive predictive value 97%) for pulmonary hypertension [8]. The average MPA/AAO diameter ratio decreases linearly from about 1.1–1.2 in infancy to < 1 in adolescence, dependent on body height [6, 7]. MPA/AAO diameter ratios < 1.4 in infants and < 1.2 in children taller than 150 cm have been shown to have a negative predictive value for pulmonary hypertension exceeding 90%.

**Figure 2:**
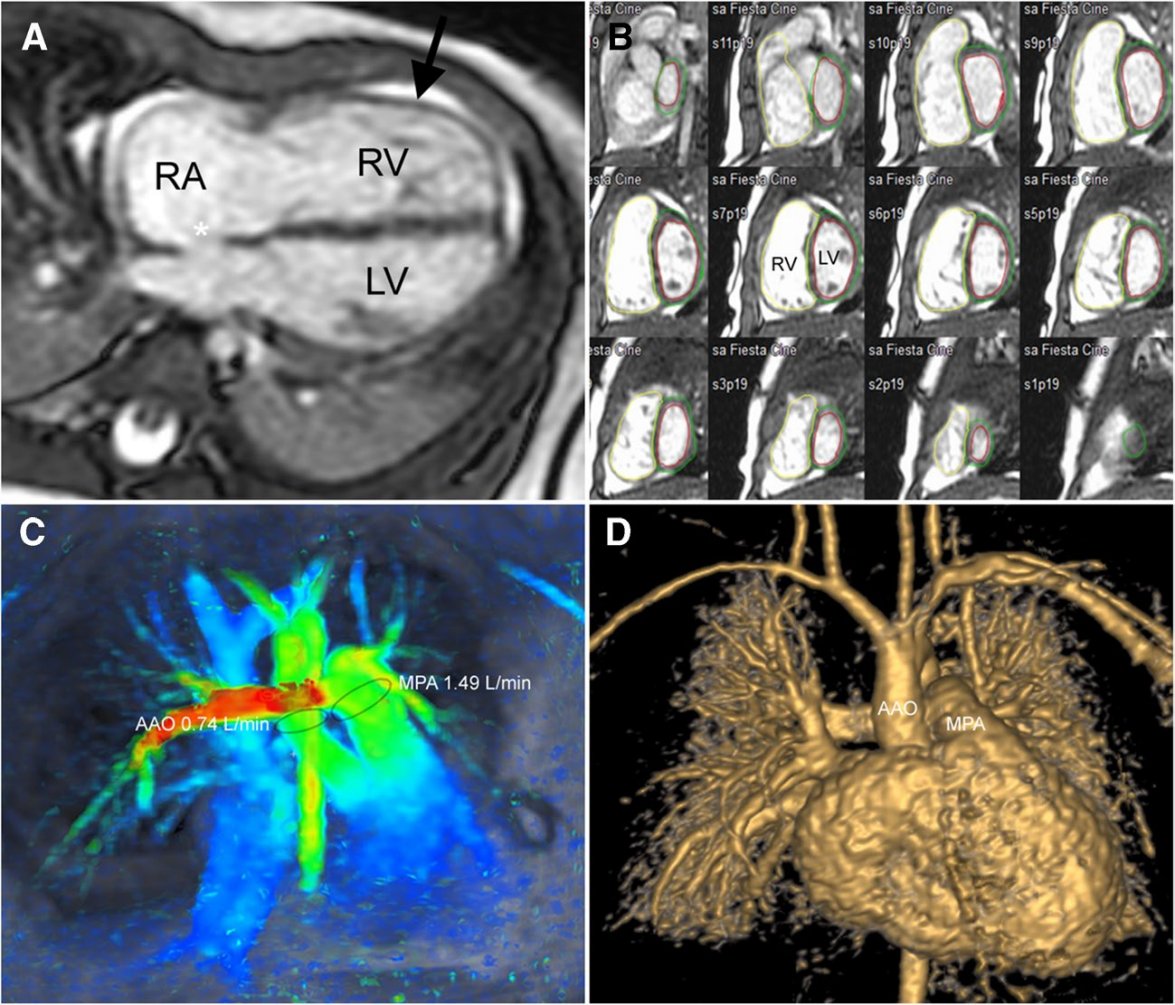
A 5-month-old girl with trisomy 21 with echocardiographic findings of pulmonary hypertension and atrial septum defect (ASD). **a** Steady-state free-precession (SSFP) cine image in the horizontal long-axis plane (4-chamber view) shows the dilated right atrium (RA) and right ventricle (RV), a normal-sized left ventricle (LV), and an atrial septum defect (*asterisk*). The right ventricular myocardium is not thickened (*arrow*). **b** Volumetry in the short-axis plane at end-diastole shows an increased right ventricular end-diastolic volume index (RV EDV 81 ml/m^2^, *z*-value 5; LV EDV 41 ml/m^2^, *z*-value -1). **c** Coronal 4-D flow MR velocity map illustrates flow measurements in the main pulmonary artery (MPA) and ascending aorta (AAO) showing increased pulmonary blood flow with *Q*_P_/*Q*_S_ = 2/1. **d** Anterior volume rendered angiographic view at peak contrast enhancement shows normal pulmonary vasculature. Transit time measured between the main pulmonary artery (MPA) and ascending aorta (AAO) is normal (3.4 s), which indicates a normal pulmonary vascular resistance

Cardiac MR remains the most accurate method for 3-dimensional (D) evaluation of the right ventricle and its dynamic interaction with the left ventricle [9]. The size of the right ventricle can be assessed qualitatively on any ECG-gated MR image obtained in the axial or horizontal long-axis planes (4-chamber view) by comparing the transverse diameters of the right and left ventricles. When there is isolated dilatation of the right ventricle, the transverse diameter of the right ventricle is larger than the diameter of the left ventricle (Figs. 1, 2). Quantitative volumetric data can be obtained from a stack of short-axis or axial cine images covering the ventricles (Fig. 2). By rule of thumb, we consider the right ventricle to be dilated when its end-diastolic volume exceeds 100 ml/m^2^ body surface area. Multicentre reference values for assessing biventricular volumes, function, and myocardial mass in children with balanced steady-state free-precession techniques (bSSFP) have recently been published [10].

Because exact measurement of the right ventricular myocardium is difficult and not very accurate due to the endocardial trabeculation, we qualitatively compare the thickness of the right ventricular anterior free wall to the thickness of the interventricular septum and left ventricular lateral wall. When the right ventricular myocardium appears as thick as the left ventricular myocardium, we consider it hypertrophied. Increased pressure in the right ventricle also leads to flattening or leftward bowing of the interventricular septum at end-systole, which is well seen in the horizontal long-axis or short-axis planes (Fig. 1).

Cardiac MR is recommended for monitoring right ventricular function in order to assess disease progression, treatment response, and prognosis [9, 11, 12]. Routine evaluation should include right ventricular ejection fraction, end-diastolic volume, stroke volume, and myocardial mass indexed to body surface area [12]. Additional MR techniques including late myocardial gadolinium enhancement or T1-mapping for quantification of myocardial fibrosis, and myocardial strain analysis for evaluation of ventricular deformation and interventricular interactions are under investigation [12–14].

## Haemodynamic magnetic resonance findings of pulmonary hypertension

The haemodynamics in the pulmonary circulation can routinely be assessed with ECG-gated velocity-encoded 2-D phase contrast (PC) techniques [15, 16] or 4-D flow MR. In a child with suspected pulmonary hypertension, flow evaluation in the main pulmonary artery (MPA), both branch pulmonary arteries (RPA and LPA), and ascending aorta (AAO) is recommended [14]. This can be achieved with a single 4-D flow acquisition covering the chest within 5–10 min (Fig. 2). If needed, any additional vessel (e.g. pulmonary veins) in the imaging volume or intracardiac flow (e.g. tricuspid valve regurgitation) can be evaluated without prolonging the study. From these flow measurements, following haemodynamic parameters should be calculated in a child with suspected pulmonary hypertension. The flow volume in the ascending aorta represents the cardiac output (*Q*_S_). Shunt flow is expressed as the pulmonary to systemic blood flow ratio (*Q*_P_/*Q*_S_ = *Q*_MPA_/*Q*_AAO_). The differential pulmonary blood flow is measured in the branch pulmonary arteries and expressed as percentage of the blood flow going to the right (*Q*_RPA_/[*Q*_RPA_ + *Q*_LPA_] × 100) and left (*Q*_LPA_/[*Q*_RPA_ + Q_LPA_] × 100) lungs [17].

The pulmonary to systemic blood flow ratio (*Q*_P_/*Q*_S_) is used to quantify an intracardiac shunt (Fig. 2). Decreased blood flow to one lung can indicate the presence of ipsilateral pulmonary vein stenosis. The cardiac output and shunt flow at baseline and during administration of pulmonary vasodilators may help guide further therapy.

Other 4-D flow findings of pulmonary hypertension include flow vortices in the dilated central pulmonary arteries, decreased average flow velocity and volumes, decreased distension and pulsatility, decreased wall shear stress, decreased kinetic energy, and altered intracardiac flow patterns [18–20]. Although it has been shown that 4-D flow parameters may correlate with pulmonary arterial pressure, clinical findings, and different subtypes of pulmonary hypertension in adults, the pathological haemodynamic flow formation in both normal and hypertensive children appears to be more variable than in adults [21]. Therefore, further study is needed for defining discriminative thresholds of 4D-flow parameters for diagnosing pulmonary hypertension or for assessing its severity in children.

Another haemodynamic parameter of the pulmonary circulation is the time interval for a contrast bolus to pass from the right-sided circulation to the left-sided circulation. In pulmonary hypertension, the increased vascular resistance results in restriction of flow through the lungs and prolongation of the pulmonary transit time. The pulmonary transit time can be measured with dynamic time-resolved contrast-enhanced techniques, which are increasingly used in clinical routine for angiography or perfusion imaging of the lungs and myocardium [22, 23]. The global and regional pulmonary transit times are significantly higher in patients with pulmonary hypertension than in controls without pulmonary hypertension [24–26]. The reported mean transit times have ranged from 4 to 10 s in studies including adults without pulmonary hypertension [27]. This highlights that the transit times through the lungs depend on the measurement method, contrast dose, rate of injection, and respiration. Reported transit times in children are scarce, ranging from 4.5 ± 0.6 s to 5.8 ± 1 s [28, 29].

**Figure 3:**
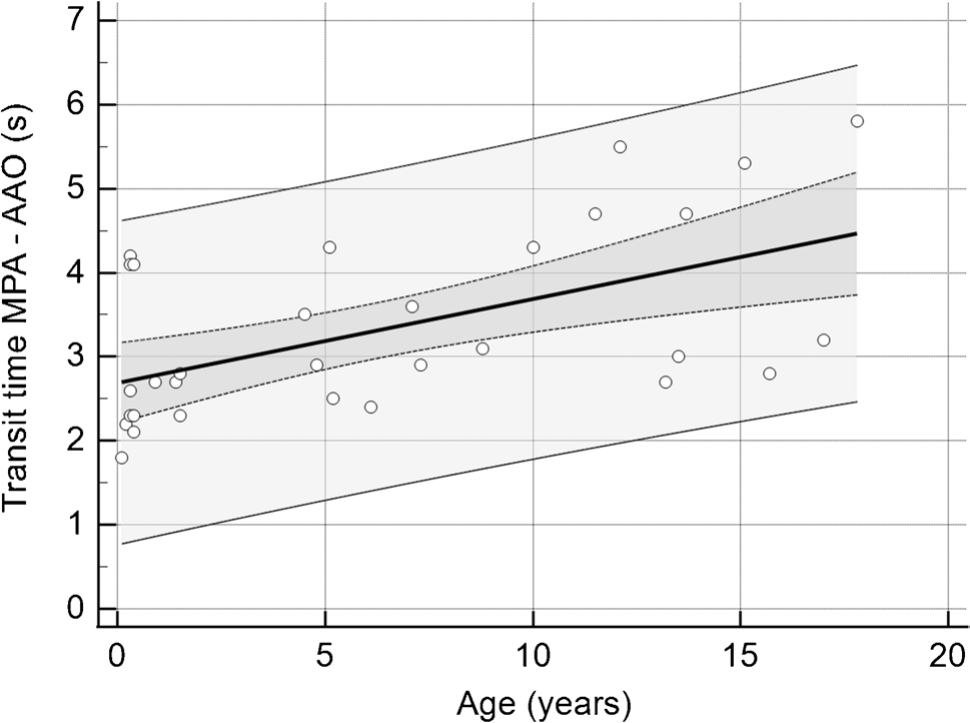
Scatterplot showing pulmonary transit times between the pulmonary trunk (MPA) and ascending aorta (AAO), obtained from dynamic contrast-enhanced time-resolved magnetic resonance angiography in 32 children without pulmonary hypertension (median age 5 years, age range 1 month to 17.8 years). Regression equation *y* = 2.6886 + 0.09991*x*, *R*^2^ 0.3085. *Dotted lines* show 95% confidence intervals and *outer lines* 95% prediction intervals

The easiest way to obtain pulmonary transit times in a clinical setting is to measure the interval between peak enhancement in the pulmonary trunk and ascending aorta during the first pass of the contrast agent. Our preliminary data show that the average time interval between the pulmonary trunk and the ascending aorta increases from less than 3 s in infancy to about 5 s in adolescence with relatively large reference intervals (Fig. 3). Our current upper limits of normal pulmonary transit times are 4.5 s in a child up to 7 years of age and 6 s in a child older than 7 years.

For lung perfusion imaging, we use a dynamic contrast-enhanced 3-D gradient echo sequence (time-resolved imaging of contrast kinetics (TRICKS)). With under-sampling of the peripheral *k*-space and by using parallel imaging, we achieve a temporal resolution of 1–2 s. Image acquisition is started together with the intravenous injection of a short bolus (2 s) of gadolinium-based contrast agent (0.1 mmol/kg body weight; gadoteric acid (Dotarem; Guerbet AG, Zürich, Switzerland)) and includes 40 phases (duration: 40–80 s).

As pulmonary transit times are directly dependent on the pulmonary vascular resistance, they may have the potential for suggesting the presence of mild pulmonary hypertension, assessing the severity of pulmonary hypertension and thus serve as predictor of long-term outcome.

## Clinical role of magnetic resonance imaging

In children, pulmonary hypertension is rare and mostly associated with congenital heart disease, a large variety of lung diseases, or it may be idiopathic [30]. There are four scenarios where MR may contribute to the evaluation of children with pulmonary hypertension.

### A child with suspected pulmonary hypertension

**Figure 4:**
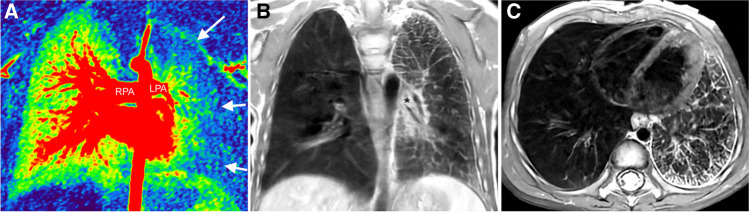
A 2-year-old boy with cystic fibrosis and occlusion of the left pulmonary veins. **a** Coronal perfusion map shows homogenous perfusion of the right lung, but no perfusion of the peripheral areas of the left lung (*arrows*). The right pulmonary artery (RPA) is dilated and the left pulmonary artery (LPA) is small. **b** Coronal and (**c**) axial proton density-weighted fast spin echo images show a small left lung with peripheral reticular interstitial pattern, pleural thickening, and ill-defined increased perihilar soft tissue (*asterisk* in (**b**)), which likely is caused by diffuse pulmonary to systemic collateral vessels

When pulmonary hypertension is suspected clinically or by echocardiography, cardiorespiratory MR can be used to exclude or demonstrate a left-to-right shunt lesion and quantify its severity by measuring the shunt volume (*Q*_P_/*Q*_S_) (Fig. 2). Recognition of a left-to-right shunt is essential, as children with patent cardiac shunts (i.e. unrecognised, unrepaired, or residual shunts) have the best survival rates [31]. A typical shunt lesion that may be recognised late is partial anomalous pulmonary venous connection (PAPVC) (Fig. 1), which often is combined with a superior sinus venosus atrial septum defect. Pulmonary vein stenosis and occlusion (Fig. 4) should be looked for with MR angiography [32]. Congenital lung malformations can be characterised and gross lung pathology (e.g. lung hypoplasia) can be excluded by adding sequences for evaluating the airways and lung parenchyma [23, 33]. Currently, the lungs are best assessed with respiratory gated proton density-weighted fast spin echo and ultra-short echo-time sequences. The presence of pulmonary oedema with interlobular septal thickening or ground-glass signal intensities should raise the concern for primary idiopathic pulmonary hypertension or pulmonary veno-occlusive disease [34, 35].

In Table 2, a typical MR protocol is given for assessing a small child with suspected pulmonary hypertension at our institution, allowing us to evaluate both the cardiovascular system and lungs in less than 20-min imaging time and without the need for breath-holding. For each MR sequence, the temporal resolution, spatial resolution, and imaging time are provided. With this protocol, the MR study can be done in sedation instead of general anaesthesia with intubation. In a child with pulmonary hypertension, breath-holding should be avoided as this can trigger a potentially lethal hypertensive crisis.

**Table 2:** Cardiovascular and lung magnetic resonance protocol for evaluating a small child with suspected or potential pulmonary hypertension at the University Children’s Hospital Zürich (1.5-T, Signa Artist; GE Healthcare, Waukesha, WI)

	SSFP localisers (FIESTA)	Cine SSFP (FIESTA)	Fast spin echo	ZTE	Dynamic time-resolved CE-MRA (TRICKS)*	4-D flow**
Information obtained	Pulmonary artery size, MPA to AAO diameter ratio, lung parenchyma (consolidation, atelectasis, pleural effusion)	Pulmonary artery size, MPA to AAO diameter ratio, (right) ventricular size, volume, and function	Lung parenchyma (ground-glass intensity, cysts, aeration, mucoid impaction)	Airways, lung parenchyma (ground-glass intensity, architectural distortion, cysts, aeration)	Lung perfusion, large vessel morphology (aorta, pulmonary arteries, pulmonary veins), pulmonary transit time	Blood flow patterns and volumes (pulmonary to systemic blood flow ratio, differential blood flow to lungs)
Orientation	Coronal Sagittal Axial	Axial Short axis (Optional: horizontal and vertical long axis)	Axial PD with fat saturation Coronal PD	Axial 3-D volume	Coronal 3-D volumes	Axial 3-D volume
Gating	No	ECG	Respiratory	Respiratory	No	ECG
Breath-hold	No	If possible	No	No	No	No
Repetition time (ms)	< 4	< 4	> 1,500	600	3.4	4.3
Echo time (ms)	1.5	1.5	40	0.0	1.3	2.3
Field of view (mm)	280	280	220	240	240	250
Spatial resolution (mm^3^)	4 × 0.5 × 0.5	4 × 0.5 × 0.5	4 × 0.4 × 0.4	1 × 1 × 1	2.5 × 0.5 × 0.5	1.6 × 1.6 × 1.6
Acquisition matrix	160 × 220	160 × 220	280 × 280	240 × 240	240 × 152	160 × 160
Temporal resolution	< 500 ms	< 25 ms	na	na	1–2 s	< 25 ms
Scan time	20–30 s per plane	1 min per stack	2–3 min per plane	3 min	30–60 s	6 min

*CE-MRA* contrast-enhanced MR angiography, *ECG* electrocardiography, *FIESTA* fast imaging employing steady-state acquisition, *PD* proton density, *SSFP* balanced steady-state free precession, *TRICKS* time-resolved imaging of contrast kinetics, *ZTE* zero echo-time

*Short bolus (2 s) of single-dose macrocyclic Gd-based contrast agent (0.1 mmol/kg body weight; gadoteric acid (Dotarem; Guerbet AG, Zürich, Switzerland))

**4-D flow MRI obtained immediately after contrast-enhanced MR angiography

### A child with a disease known to be associated with pulmonary hypertension

**Figure 5:**
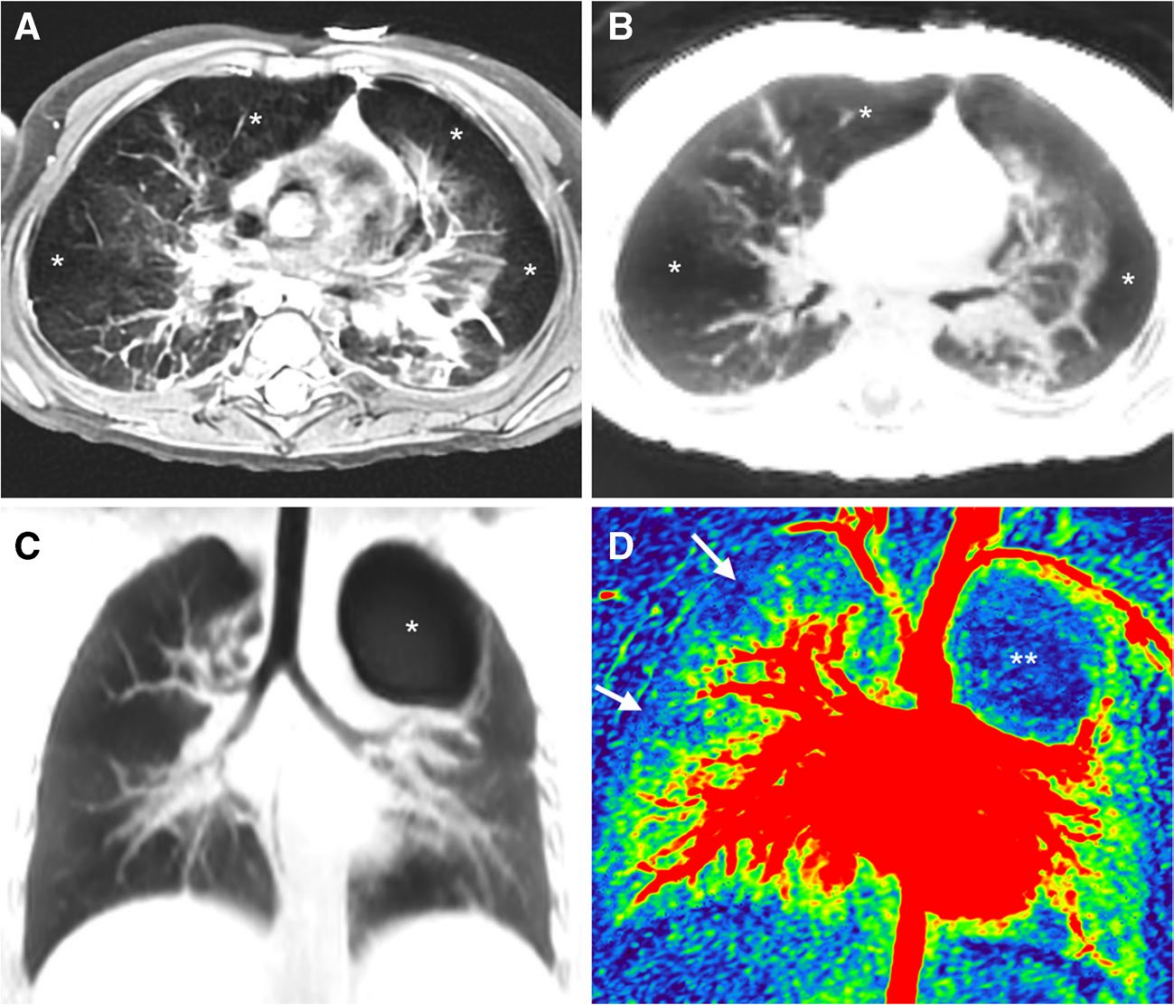
A 2-year-old boy with severe bronchopulmonary dysplasia. **a** Axial proton density-weighted fast spin echo and (**b**) axial zero echo time images show architectural distortion with atelectatic areas side by side with hyperinflated areas (*asterisk*). **c** Coronal zero echo time image shows the normal trachea and central bronchi, mosaic pattern of lung signal intensity, and an air-filled cyst (*asterisk*). **d** Coronal perfusion map shows no perfusion in the air-filled cyst (*asterisk*) and decreased perfusion in the periphery of the right upper lobe (*arrows*)

**Figure 6:**
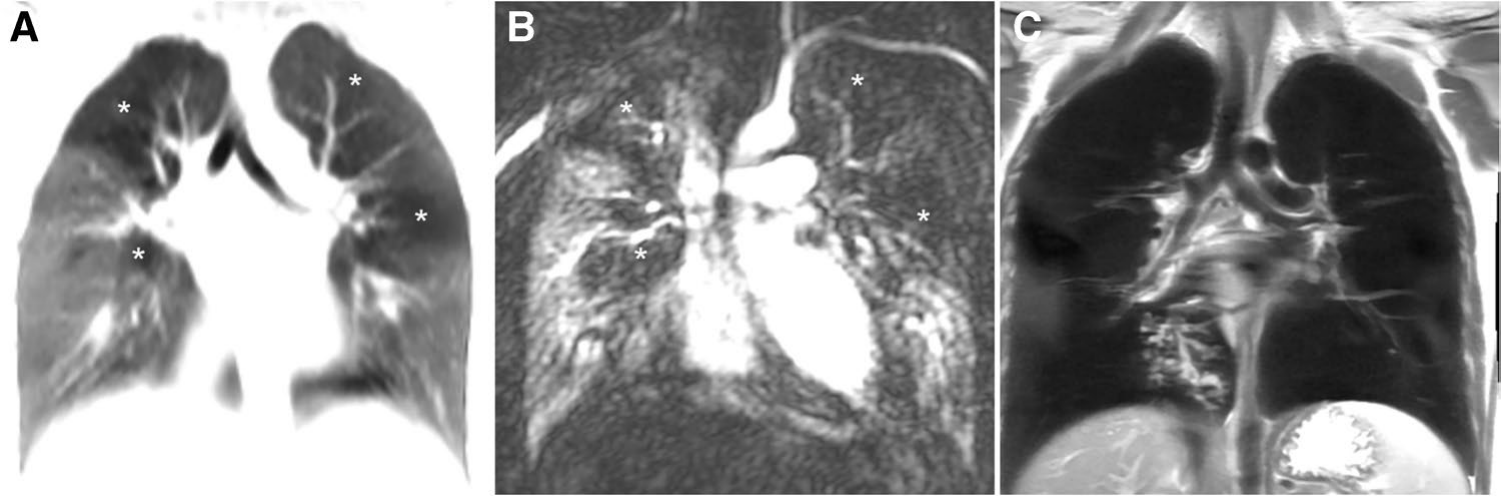
A 12-year-old boy with bronchiolitis obliterans. **a** Coronal zero echo time image shows mosaic signal intensity with decreased lung signal in both upper lobes (*asterisk*). **b** Coronal subtraction angiographic image at peak lung enhancement shows corresponding perfusion defects (*asterisk*). **c** Coronal perfusion map shows corresponding decreased blood volume (*asterisk*). The pulmonary transit time between the pulmonary trunk and ascending aorta is normal (3 s), which indicates a normal pulmonary vascular resistance

**Figure 7:**
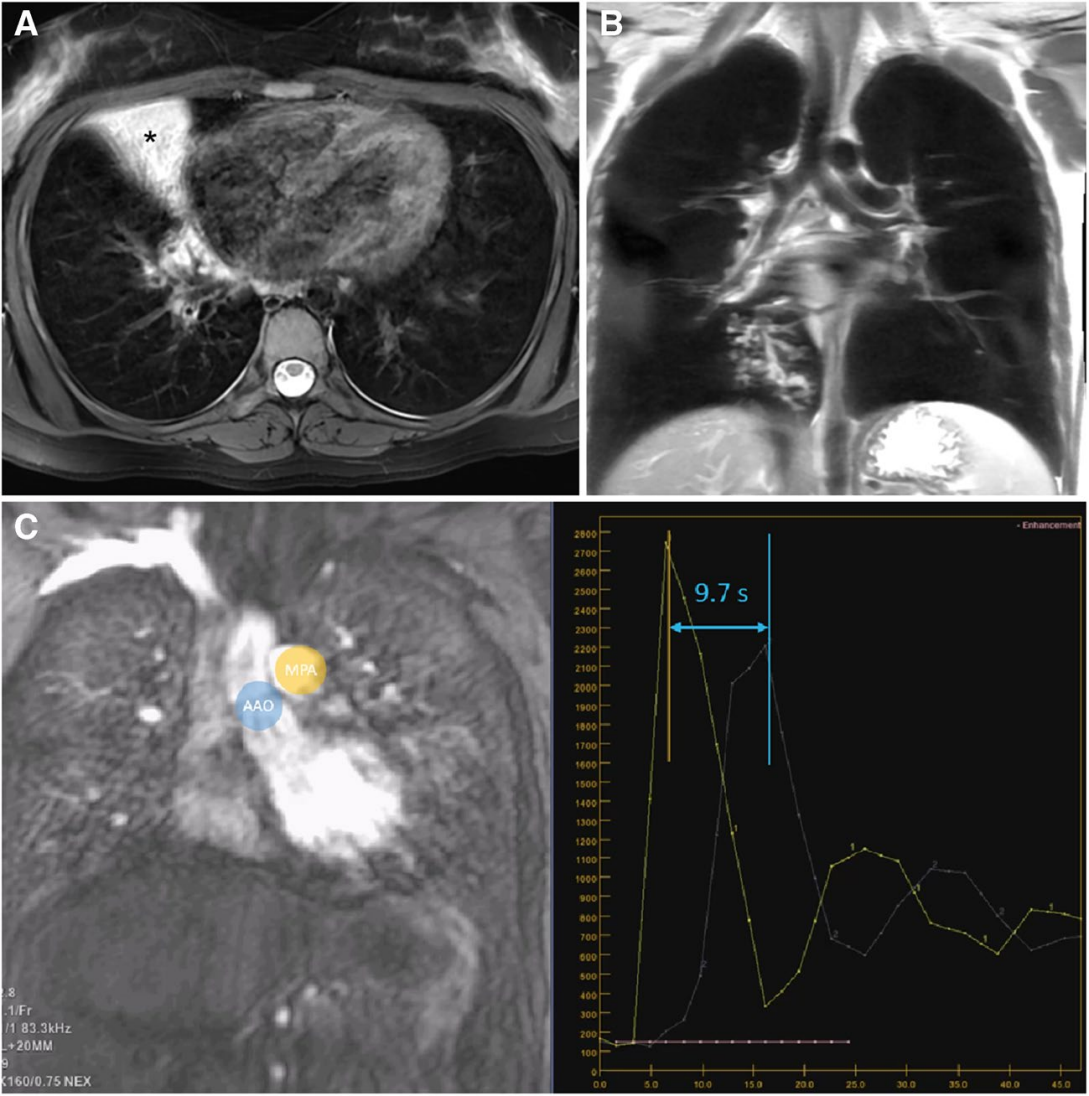
A 17-year-old female with primary ciliary dyskinesia. **a** Axial respiratory gated T2-weighted fast spin echo image shows characteristic consolidation (*asterisk*) in the middle lobe and bronchial wall thickening of lower lobe segmental bronchi. **b** Coronal respiratory gated proton density-weighted fast spin echo image shows mucus plugging and tree-in-bud formations in the medial basal segment. **c** Coronal angiographic image and signal-time curves from the pulmonary trunk (MPA, *yellow region of interest and curve*) and ascending aorta (AAO, *blue region of interest and curve*) illustrate measurement of the transit time between peak enhancement of the MPA and AAO. The pulmonary transit time is prolonged (9.7 s), which indicates an increased pulmonary vascular resistance

Common lung diseases associated with pulmonary hypertension include bronchopulmonary dysplasia (Fig. 5) and lung growth abnormalities in infants, as well as any chronic lung disease or obstructive sleep apnoea in older children [30]. At our centre, lung MR has been established more than a decade ago as part of routine care in children with cystic fibrosis, primary ciliary dyskinesia (Fig. 6), and bronchopulmonary dysplasia. Currently, it is becoming the method of choice when looking for any small airways disease including bronchiolitis obliterans (Fig. 7). As most of our dedicated MR studies are performed in children who potentially could have pulmonary hypertension, we scrutinize every lung MR for the respective MR signs. In children with bronchopulmonary dysplasia, we carefully look for associated pulmonary vein stenosis. When the central pulmonary arteries are enlarged with an MPA/AAO diameter ratio > 1.2 in an infant and > 1.1 in an older child, and/or the pulmonary transit time obtained from time-resolved contrast-enhanced MR angiography is prolonged, we suggest further investigation by echocardiography.

### A child with a chest magnetic resonance study performed for any indication

In any imaging study of the chest, enlargement of the central pulmonary arteries and right heart should raise the possibility of pulmonary hypertension. When signs of pulmonary hypertension are identified, the clinical colleagues should be alerted to the high probability of hypertension, so that further investigations such as echocardiography or right-heart catheterisation can be pursued if clinically indicated.

### Longitudinal monitoring of children with pulmonary hypertension

Cardiac MR is the most accurate imaging method for monitoring right ventricular volumes and function in order to assess disease progression, treatment response, and prognosis. Pulmonary transit times obtained from dynamic contrast-enhanced MR angiography have the potential to non-invasively provide longitudinal data on pulmonary vascular resistance.

## Summary

Recognition of structural cardiovascular and haemodynamic findings of pulmonary hypertension on chest MR may identify patients with unexpected preclinical pulmonary hypertension. Dedicated cardiovascular and pulmonary MR may contribute to the diagnosis of the underlying causative disease. Assessment of the pulmonary transit time and ventricular function may contribute to the detection and monitoring of pulmonary hypertension in children. An advantage of MR is that it can provide morphological and functional information of the heart, pulmonary circulation, and lung parenchyma in a single examination within a reasonably short imaging time.

## Data Availability

No datasets were generated or analysed during the current study.

## References

[1] Simonneau G, Montani D, Celermajer DS, Denton CP, Gatzoulis MA, Krowka M, Williams PG, Souza R (2019) Haemodynamic definitions and updated clinical classification of pulmonary hypertension. Eur Respir J. 53:1801913 10.1183/13993003.01913-2018 PMC6351336 30545968

[2] Sirajuddin A, Mirmomen SM, Henry TS, Kandathil A, Kelly AM, King CS, Kuzniewski CT, Lai AR, Lee E, Martin MD, Mehta P, Morris MF, Raptis CA, Roberge EA, Sandler KL, Donnelly EF (2022) ACR Appropriateness Criteria® suspected pulmonary hypertension: 2022 update. J Am Coll Radiol 19:S502-s512 36436973 10.1016/j.jacr.2022.09.018

[3] Dong TX, Zhu Q, Wang ST, Wang YH, Li GY, Kong FX, Ma CY (2023) Diagnostic and prognostic value of echocardiography in pulmonary hypertension: an umbrella review of systematic reviews and meta-analyses. BMC Pulm Med 23:253 37430308 10.1186/s12890-023-02552-y PMC10334642

[4] Ng CS, Wells AU, Padley SP (1999) A CT sign of chronic pulmonary arterial hypertension: the ratio of main pulmonary artery to aortic diameter. J Thorac Imaging 14:270–278 10524808 10.1097/00005382-199910000-00007

[5] Knobel Z, Kellenberger CJ, Kaiser T, Albisetti M, Bergstrasser E, Buechel ER (2011) Geometry and dimensions of the pulmonary artery bifurcation in children and adolescents: assessment in vivo by contrast-enhanced MR-angiography. Int J Cardiovasc Imaging 27:385–396 20652636 10.1007/s10554-010-9672-6

[6] Chen SJ, Huang JH, Lee WJ, Lin MT, Chen YS, Wang JK (2019) Diagnosis of pulmonary arterial hypertension in children by using cardiac computed tomography. Korean J Radiol 20:976–984 31132823 10.3348/kjr.2018.0673 PMC6536789

[7] Compton GL, Florence J, MacDonald C, Yoo SJ, Humpl T, Manson D (2015) Main pulmonary artery-to-ascending aorta diameter ratio in healthy children on MDCT. AJR Am J Roentgenol 205:1322–1325 26587940 10.2214/AJR.15.14301

[8] Caro-Domínguez P, Compton G, Humpl T, Manson DE (2016) Pulmonary arterial hypertension in children: diagnosis using ratio of main pulmonary artery to ascending aorta diameter as determined by multi-detector computed tomography. Pediatr Radiol 46:1378–1383 27194438 10.1007/s00247-016-3636-5

[9] Kiely DG, Levin D, Hassoun P, Ivy DD, Jone PN, Bwika J, Kawut SM, Lordan J, Lungu A, Mazurek J, Moledina S, Olschewski H, Peacock A, Puri GD, Rahaghi F, Schafer M, Schiebler M, Screaton N, Tawhai M, Van Beek EJ, Vonk-Noordegraaf A, Vanderpool RR, Wort J, Zhao L, Wild J, Vogel-Claussen J, Swift AJ (2019) EXPRESS: statement on imaging and pulmonary hypertension from the Pulmonary Vascular Research Institute (PVRI). Pulm Circ 9:2045894019841990 30880632 10.1177/2045894019841990 PMC6732869

[10] van der Ven JPG, Sadighy Z, Valsangiacomo Buechel ER, Sarikouch S, Robbers-Visser D, Kellenberger CJ, Kaiser T, Beerbaum P, Boersma E, Helbing WA (2020) Multicentre reference values for cardiac magnetic resonance imaging derived ventricular size and function for children aged 0–18 years. Eur Heart J Cardiovasc Imaging 21:102–113 31280290 10.1093/ehjci/jez164 PMC6923680

[11] Moledina S, Pandya B, Bartsota M, Mortensen KH, McMillan M, Quyam S, Taylor AM, Haworth SG, Schulze-Neick I, Muthurangu V (2013) Prognostic significance of cardiac magnetic resonance imaging in children with pulmonary hypertension. Circ Cardiovasc Imaging 6:407–414 23572488 10.1161/CIRCIMAGING.112.000082

[12] Latus H, Kuehne T, Beerbaum P, Apitz C, Hansmann G, Muthurangu V, Moledina S (2016) Cardiac MR and CT imaging in children with suspected or confirmed pulmonary hypertension/pulmonary hypertensive vascular disease. Expert consensus statement on the diagnosis and treatment of paediatric pulmonary hypertension. The European Paediatric Pulmonary Vascular Disease Network, endorsed by ISHLT and DGPK. Heart 102:30–35 10.1136/heartjnl-2015-308246 27053695

[13] Alabed S, Garg P, Johns CS, Alandejani F, Shahin Y, Dwivedi K, Zafar H, Wild JM, Kiely DG, Swift AJ (2020) Cardiac magnetic resonance in pulmonary hypertension-an update. Curr Cardiovasc Imaging Rep 13:30 33184585 10.1007/s12410-020-09550-2 PMC7648000

[14] Latus H, Meierhofer C (2021) Role of cardiovascular magnetic resonance in pediatric pulmonary hypertension-novel concepts and imaging biomarkers. Cardiovasc Diagn Ther 11:1057–1069 34527532 10.21037/cdt-20-270 PMC8410495

[15] Kellenberger CJ, Yoo SJ, Buchel ER (2007) Cardiovascular MR imaging in neonates and infants with congenital heart disease. Radiographics 27:5–18 17234995 10.1148/rg.271065027

[16] Kellenberger CJ, Macgowan CK, Roman KS, Al-Habshan F, Benson LN, Redington AN, Yoo SJ (2005) Hemodynamic evaluation of the peripheral pulmonary circulation by cine phase-contrast magnetic resonance imaging. J Magn Reson Imaging 22:780–787 16270288 10.1002/jmri.20447

[17] Roman KS, Kellenberger CJ, Farooq S, MacGowan CK, Gilday DL, Yoo SJ (2005) Comparative imaging of differential pulmonary blood flow in patients with congenital heart disease: magnetic resonance imaging versus lung perfusion scintigraphy. Pediatr Radiol 35:295–301 15490145 10.1007/s00247-004-1344-z

[18] Zhao X, Leng S, Tan RS, Chai P, Yeo TJ, Bryant JA, Teo LLS, Fortier MV, Ruan W, Low TT, Ong CC, Zhang S, van der Geest RJ, Allen JC, Hughes M, Garg P, Tan TH, Yip JW, Tan JL, Zhong L (2022) Right ventricular energetic biomarkers from 4D flow CMR are associated with exertional capacity in pulmonary arterial hypertension. J Cardiovasc Magn Reson 24:61 36451198 10.1186/s12968-022-00896-8 PMC9714144

[19] Cain MT, Schäfer M, Ross LK, Ivy DD, Mitchell MB, Fenster BE, Bull TM, Barker AJ, Vargas D, Hoffman JRH (2023) 4D-Flow MRI intracardiac flow analysis considering different subtypes of pulmonary hypertension. Pulm Circ 13:e12307 37941938 10.1002/pul2.12307 PMC10628368

[20] Ota H, Kamada H, Higuchi S, Takase K (2022) Clinical application of 4D flow MR imaging to pulmonary hypertension. Magn Reson Med Sci 21:309–318 35185084 10.2463/mrms.rev.2021-0111 PMC9680544

[21] Schäfer M, Ivy DD, Abman SH, Stenmark K, Browne LP, Barker AJ, Mitchell MB, Morgan GJ, Wilson N, Shah A, Kollengode M, Naresh N, Fonseca B, DiMaria M, Buckner JK, Hunter KS, Kheyfets V, Fenster BE, Truong U (2019) Differences in pulmonary arterial flow hemodynamics between children and adults with pulmonary arterial hypertension as assessed by 4D-flow CMR studies. Am J Physiol Heart Circ Physiol 316:H1091-h1104 30822118 10.1152/ajpheart.00802.2018 PMC7327229

[22] Buechel ER, Balmer C, Bauersfeld U, Kellenberger CJ, Schwitter J (2009) Feasibility of perfusion cardiovascular magnetic resonance in paediatric patients. J Cardiovasc Magn Reson 11:51 19948020 10.1186/1532-429X-11-51 PMC2789062

[23] Kellenberger CJ, Amaxopoulou C, Moehrlen U, Bode PK, Jung A, Geiger J (2020) Structural and perfusion magnetic resonance imaging of congenital lung malformations. Pediatr Radiol 50:1083–1094 32303778 10.1007/s00247-020-04658-5 PMC7329781

[24] Ley S, Fink C, Zaporozhan J, Borst MM, Meyer FJ, Puderbach M, Eichinger M, Plathow C, Grünig E, Kreitner KF, Kauczor HU (2005) Value of high spatial and high temporal resolution magnetic resonance angiography for differentiation between idiopathic and thromboembolic pulmonary hypertension: initial results. Eur Radiol 15:2256–2263 16041529 10.1007/s00330-005-2792-z

[25] Jeong HJ, Vakil P, Sheehan JJ, Shah SJ, Cuttica M, Carr JC, Carroll TJ, Davarpanah A (2011) Time-resolved magnetic resonance angiography: evaluation of intrapulmonary circulation parameters in pulmonary arterial hypertension. J Magn Reson Imaging 33:225–231 21182144 10.1002/jmri.22428 PMC3059715

[26] Moore JE, Cerne JW, Pathrose A, Veer M, Sarnari R, Ragin A, Carr JC, Markl M (2023) Quantitative assessment of regional pulmonary transit times in pulmonary hypertension. J Magn Reson Imaging 57:727–737 35808987 10.1002/jmri.28343

[27] Edwards L, Waterton JC, Naish J, Short C, Semple T, Jm Parker G, Tibiletti M (2023) Imaging human lung perfusion with contrast media: a meta-analysis. Eur J Radiol 164:110850 37178490 10.1016/j.ejrad.2023.110850

[28] Macgowan CK, Al-Kwifi O, Varodayan F, Yoo SJ, Wright GA, Kellenberger CJ (2005) Optimization of 3D contrast-enhanced pulmonary magnetic resonance angiography in pediatric patients with congenital heart disease. Magn Reson Med 54:207–212 15968668 10.1002/mrm.20538

[29] Groß V, Zahn K, Maurer K, Wessel L, Schaible T, Schoenberg SO, Weiß C, Zoellner FG, Weis M (2022) MR lung perfusion measurements in adolescents after congenital diaphragmatic hernia: correlation with spirometric lung function tests. Eur Radiol 32:2572–2580 34741621 10.1007/s00330-021-08315-9 PMC8921025

[30] Sullivan RT, Raj JU, Austin ED (2023) Recent advances in pediatric pulmonary hypertension: implications for diagnosis and treatment. Clin Ther 45:901–912 37517916 10.1016/j.clinthera.2023.07.001

[31] Ploegstra MJ, Ivy DD, Beghetti M, Bonnet D, Alehan D, Ablonczy L, Mattos S, Bowers D, Humpl T, Berger RMF (2024) Long-term outcome of children with newly diagnosed pulmonary arterial hypertension: results from the Global TOPP registry. Eur Heart J Qual Care Clin Outcomes 10:66–76 36972621 10.1093/ehjqcco/qcad020 PMC10785586

[32] Valsangiacomo ER, Levasseur S, McCrindle BW, MacDonald C, Smallhorn JF, Yoo SJ (2003) Contrast-enhanced MR angiography of pulmonary venous abnormalities in children. Pediatr Radiol 33:92–98 12557064 10.1007/s00247-002-0789-1

[33] Kellenberger CJ, Lovrenski J, Semple T, Caro-Dominguez P (2023) Neonatal cardiorespiratory imaging-a multimodality state-of-the-art review. Pediatr Radiol 53:660–676 36138217 10.1007/s00247-022-05504-6

[34] Semple T, Winant AJ, Lee EY (2022) Childhood interstitial lung disease: imaging guidelines and recommendations. Radiol Clin North Am 60:83–111 34836568 10.1016/j.rcl.2021.08.009

[35] Pfluger M, Humpl T (2021) Pulmonary veno-occlusive disease in childhood-a rare disease not to be missed. Cardiovasc Diagn Ther 11:1070–1079 34527533 10.21037/cdt-20-320 PMC8410504

